# Supplementary figures and images for: Variability of ACOX1 Gene Polymorphisms across Different Horse Breeds with Regard to Selection Pressure
Source: Animals (Basel). 2020 Nov 27;10(12):2225. doi: 10.3390/ani10122225 (PMC7761022; doi:10.3390/ani10122225)

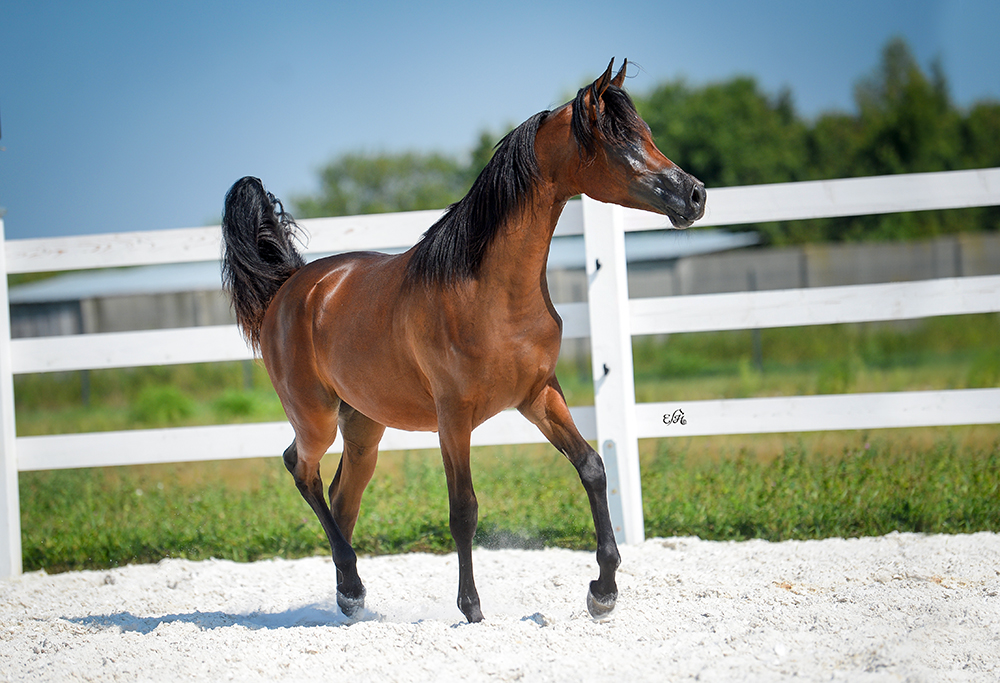

Supplement: Supplementary file 1 [file animals-10-02225-s001.zip › animals-994193-supplementary/Supplementary v2/Sup file 1 The Arabian horse (photo credit Ewa Imielska-Hebda all rights reserved).jpg]

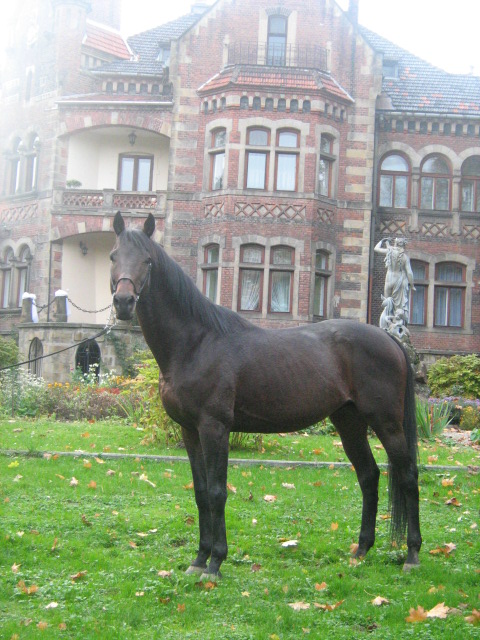

Supplement: Supplementary file 1 [file animals-10-02225-s001.zip › animals-994193-supplementary/Supplementary v2/Sup file 2 Thoroughbred (photo credit Bogusława Długosz; all rights reserved).jpg]

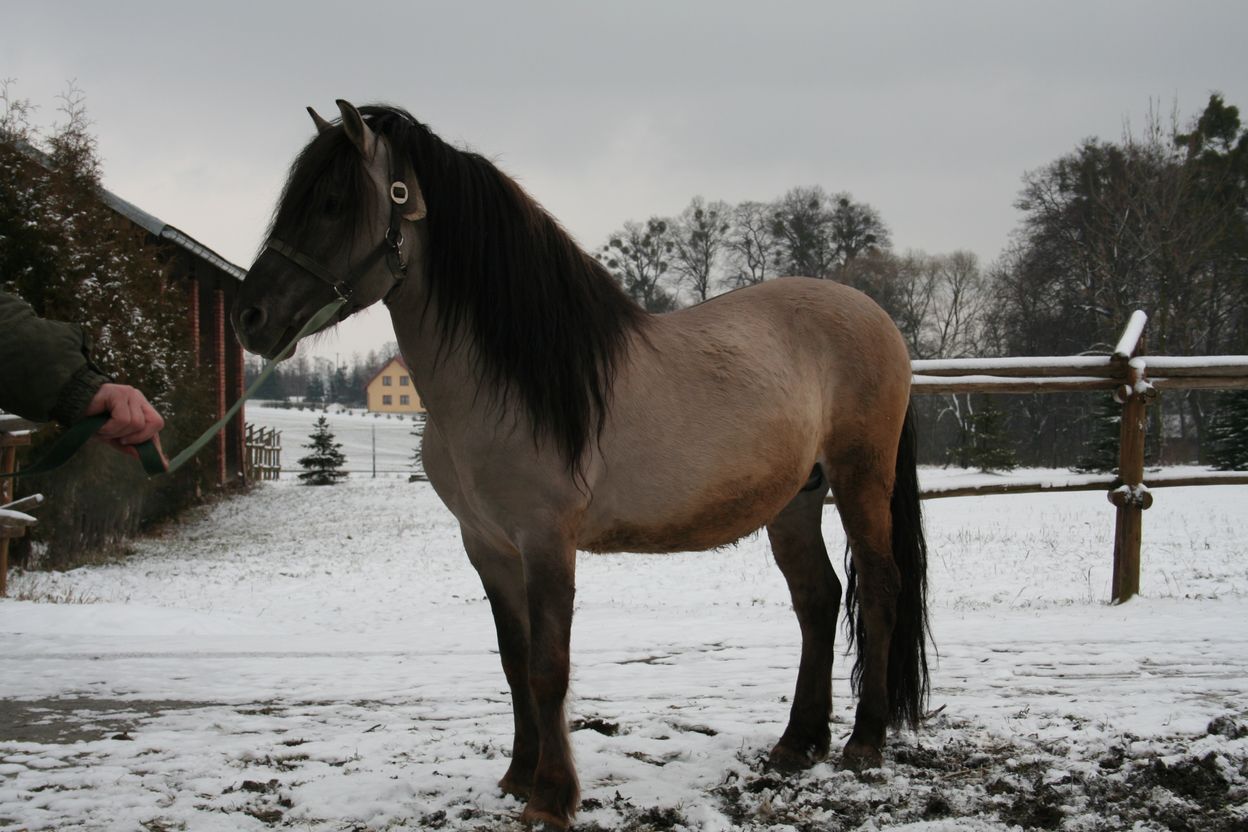

Supplement: Supplementary file 1 [file animals-10-02225-s001.zip › animals-994193-supplementary/Supplementary v2/Sup file 3 Polish Konik (photo credit Bogusława Długosz; all rights reserved).JPG]

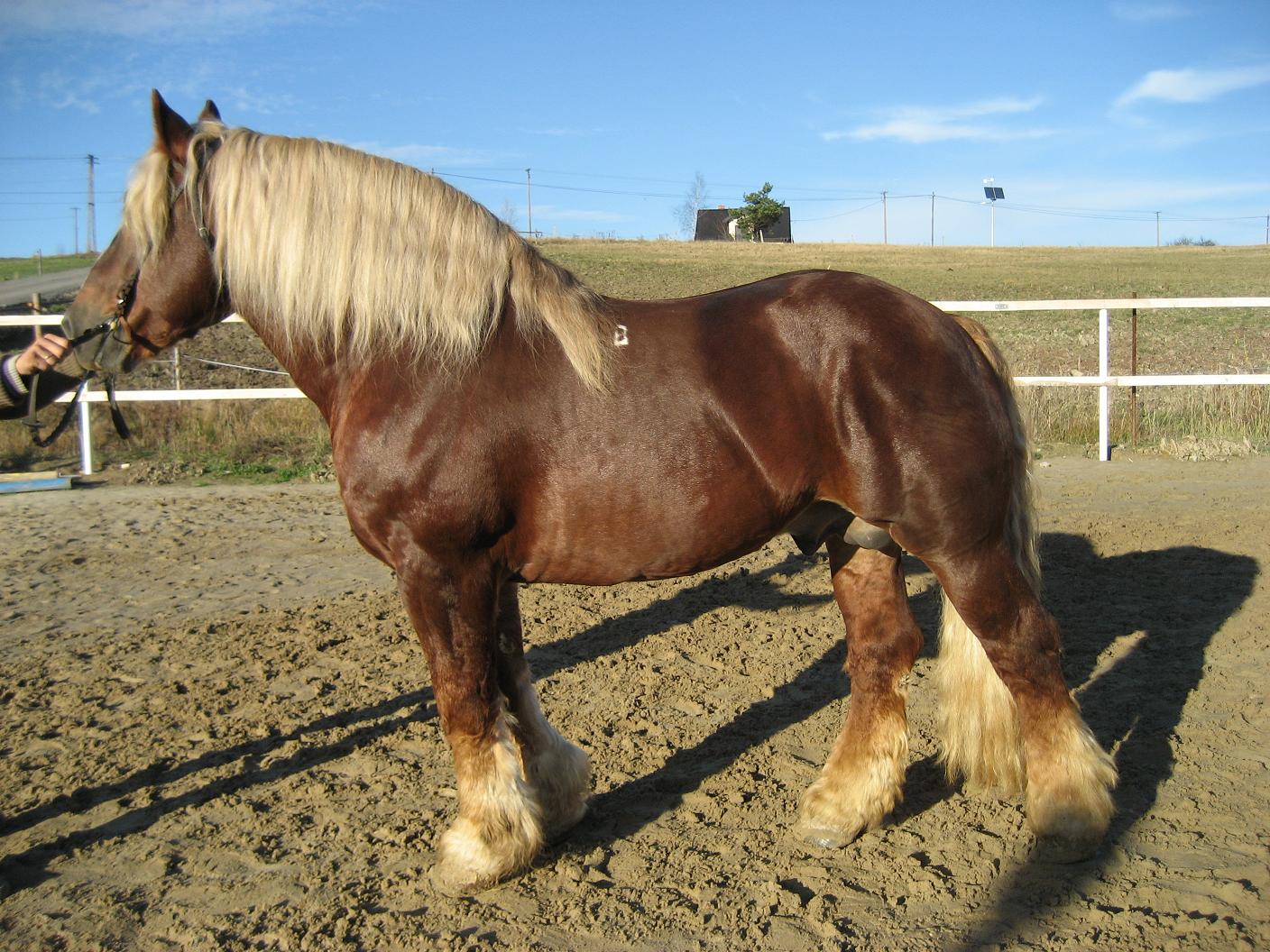

Supplement: Supplementary file 1 [file animals-10-02225-s001.zip › animals-994193-supplementary/Supplementary v2/Sup file 4 Draft horse (photo credit Bogusława Długosz; all rights reserved).jpg]

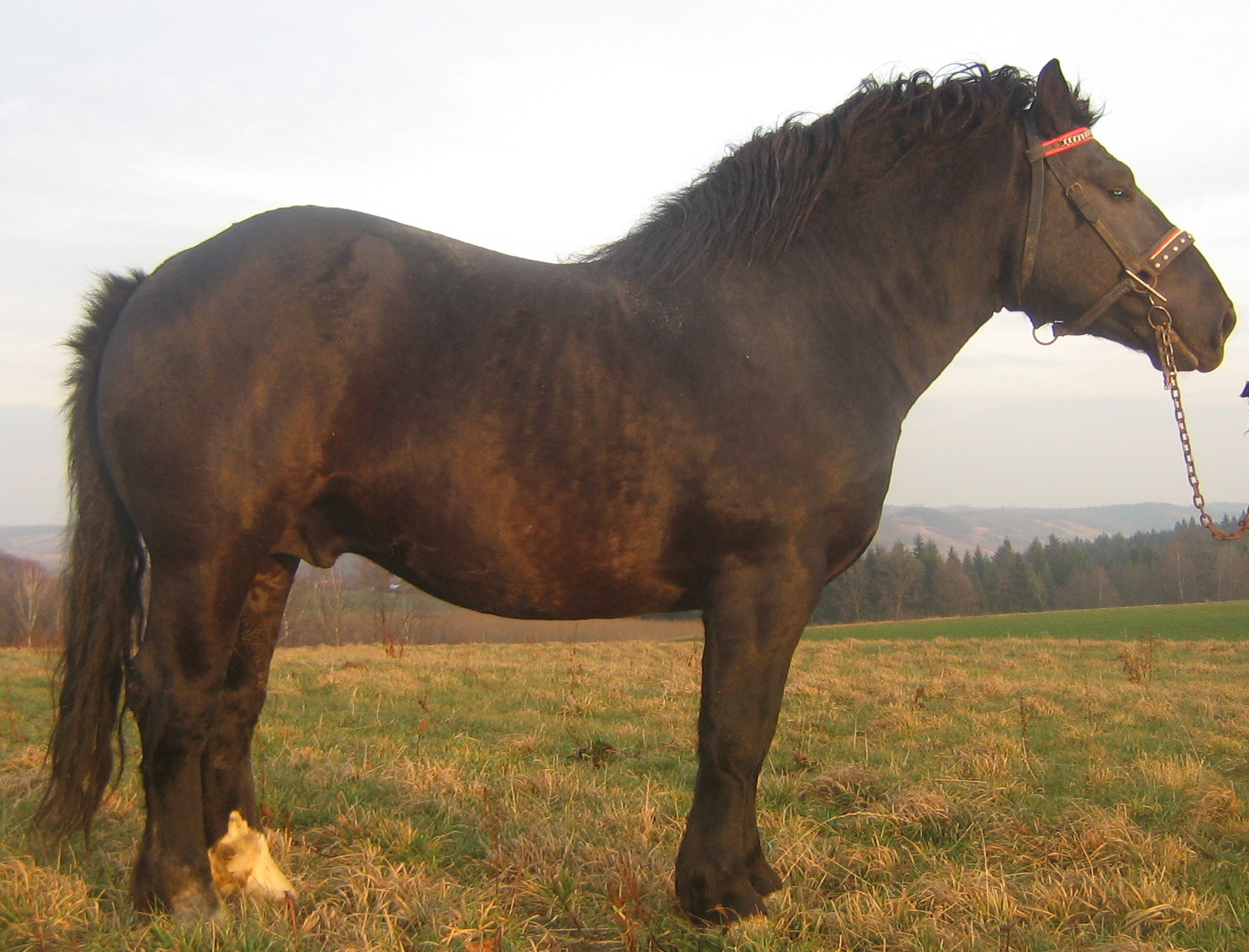

Supplement: Supplementary file 1 [file animals-10-02225-s001.zip › animals-994193-supplementary/Supplementary v2/Sup file 5 Hucul (photo credit Bogusława Długosz; all rights reserved).JPG]

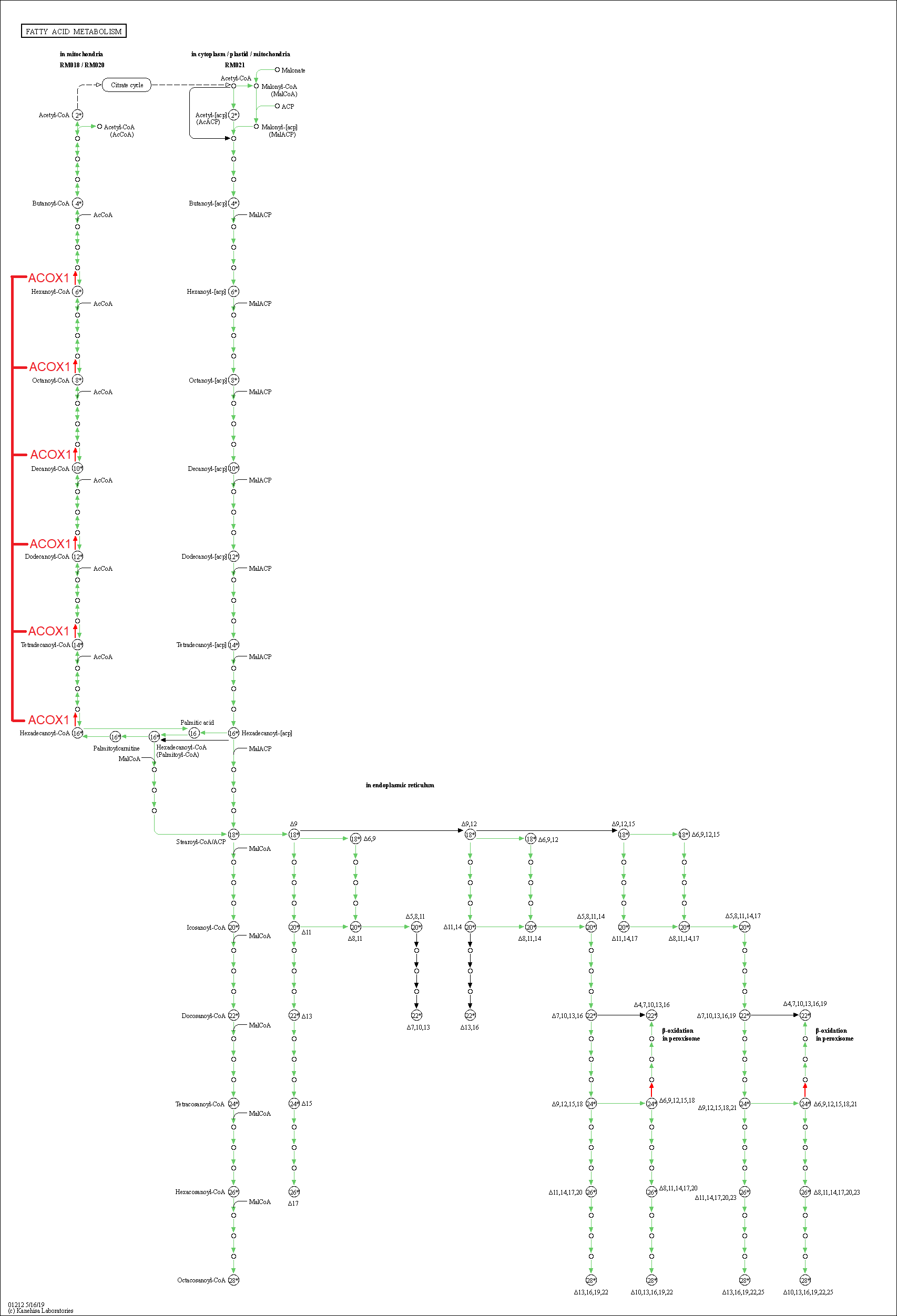

Supplement: Supplementary file 1 [file animals-10-02225-s001.zip › animals-994193-supplementary/Supplementary v2/Sup file 6 The fatty acid metabolism with beta-oxidation module based on KEGG Equus caballus reference(ecb01212; M00087).png]

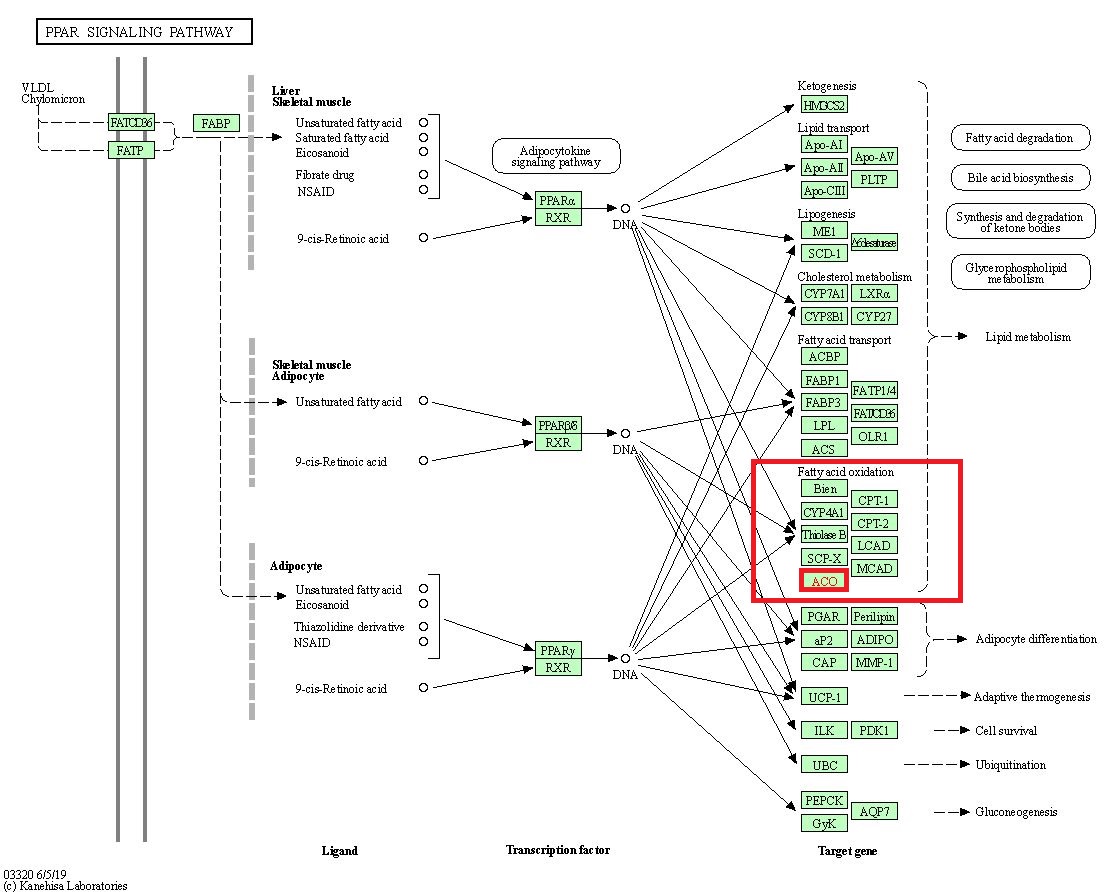

Supplement: Supplementary file 1 [file animals-10-02225-s001.zip › animals-994193-supplementary/Supplementary v2/Sup file 7 The PPAR signalling pathways based on KEGG Equus caballus reference (ecb03320) .JPG]
